# P-2145. *Candida blankii*: An Emerging Threat among Hospitalized Neonates of Bangladesh

**DOI:** 10.1093/ofid/ofae631.2299

**Published:** 2025-01-29

**Authors:** Tanzir Ahmed Shuvo, Nusrat Jahan Shaly, Farhat Jaby Pammi, Md Kamrul Islam, Adiba Tasnim, Shams E Tabriz Bhuiyan, Fahmida Dil Farzana, Md Abdul Aleem, Fahmida Chowdhury, Sayeeda Huq

**Affiliations:** icddr,b, Dhaka, Dhaka, Bangladesh; iccdr,b, Mohakhali, Dhaka, Bangladesh; icddr,b, Dhaka, Dhaka, Bangladesh; icddr,b, Dhaka, Dhaka, Bangladesh; icddr,b, Dhaka, Dhaka, Bangladesh; icddr,b, Dhaka, Dhaka, Bangladesh; icddr,b, Dhaka, Dhaka, Bangladesh; icddr,b, Dhaka, Dhaka, Bangladesh; icddr,b, Dhaka, Dhaka, Bangladesh; icddr,b, Dhaka, Dhaka, Bangladesh

## Abstract

**Background:**

Invasive fungal infections (IFIs) cause serious complications and fatality. Despite the severity of such magnitude, the true burden of these infections remains unclear. In LMICs like Bangladesh, there is a lack of surveillance mechanisms to understand the burden of IFIs. *Candida blankii* (*C. blankii*) is an emerging clinical pathogen with first reported human infection in 2014 and limited global reports of clinical infection since then. This study aims to investigate the burden on *C. blankii* along with other IFIs among hospitalized neonates in Bangladesh.

**Figure:**
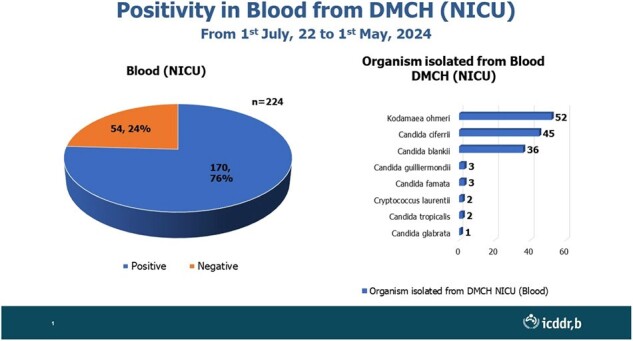

**Methods:**

Since July 2022, a hospital-based IFI surveillance has been ongoing in the neonatal ICU of a tertiary level hospital in Bangladesh. From then until April 2024, 224 patients were enrolled, who were suspected of have IFIs based on predefined inclusion and exclusion criteria. Blood and endotracheal aspirate samples were collected and sent to the laboratory for microscopy, culture and identification using Vitek 2 and Vitek MS automated system. Additionally, antifungal susceptibility testing (AFST) was performed using the Vitek2 system.

**Figure:**
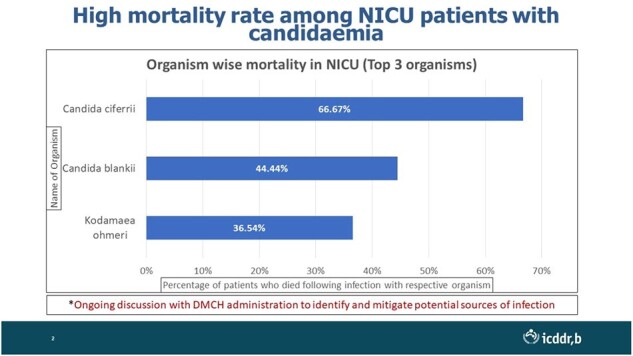

**Results:**

Median age of the enrolled neonates were 17 days (IQR:12-21); 67% of them were male. Notably, 76% of these neonates had positive blood cultures for at least one fungal organism. *Kodamaea ohmeri* was the most frequently identified fungus (52 cases), followed by C*andida cifferi* (45) and the emerging fungus *C. blankii* (36). There were only two cases of *Cryptococcus laurentii* along with other candida species. Mortality rates were highest among neonates infected with *Candida cifferi* (67%), followed by *C. blankii* (45%) and *Kodamaea ohmeri* (36%). Worryingly, C. *blankii* exhibited high resistance to Amphotericin B (97%) and Voriconazole (69%), potentially limiting effective treatment options.

High Antifungal Resistance

**Figure:**
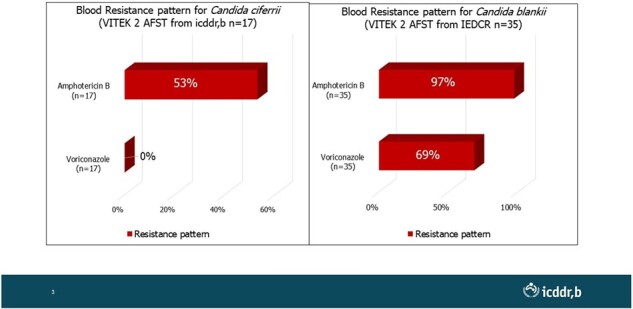

**Conclusion:**

This ongoing surveillance identified a high prevalence of IFIs, particularly caused by the emerging fungus *C. blankii*. Further research is necessary to elucidate the potential sources and transmission pathways of *C. blankii* to design targeted interventions to effectively control this emerging threat in this vulnerable population. The surveillance team is actively working with the hospital authorities to identify potential sources and implement strategies to minimize such infections.

**Disclosures:**

All Authors: No reported disclosures

